# Farnesyltransferase inhibitor lonafarnib suppresses respiratory syncytial virus infection by blocking conformational change of fusion glycoprotein

**DOI:** 10.1038/s41392-024-01858-5

**Published:** 2024-06-10

**Authors:** Qi Yang, Bao Xue, Fengjiang Liu, Yongzhi Lu, Jielin Tang, Mengrong Yan, Qiong Wu, Ruyi Chen, Anqi Zhou, Lijie Liu, Junjun Liu, Changbin Qu, Qingxin Wu, Muqing Fu, Jiayi Zhong, Jianwei Dong, Sijie Chen, Fan Wang, Yuan Zhou, Jie Zheng, Wei Peng, Jinsai Shang, Xinwen Chen

**Affiliations:** 1 Guangzhou National Laboratory, Guangzhou, 510005 China; 2 State Key Laboratory of Respiratory Disease, Guangzhou Medical University, Guangzhou, 511436 China; 3 Wuhan Institute of Virology, Chinese Academy of Sciences, Wuhan, 430071 China; 4 School of Basic Medical Sciences, Guangzhou Medical University, Guangzhou, 511436 China; 5 GMU-GIBH Joint School of Life Sciences, Guangzhou Medical University, Guangzhou, 511436 China; 6 Guangzhou Institutes of Biomedicine and Health, Chinese Academy of Sciences, Guangzhou, 510530 China; 7 Shanghai Institute of Materia Medica, Chinese Academy of Sciences, Shanghai, 201203 China; 8 School of Pharmaceutical Science and Technology, Hangzhou lnstitute for Advanced Study, UCAS, Hangzhou, 310024 China

**Keywords:** Target validation, Structural biology

## Abstract

Respiratory syncytial virus (RSV) is the major cause of bronchiolitis and pneumonia in young children and the elderly. There are currently no approved RSV-specific therapeutic small molecules available. Using high-throughput antiviral screening, we identified an oral drug, the prenylation inhibitor lonafarnib, which showed potent inhibition of the RSV fusion process. Lonafarnib exhibited antiviral activity against both the RSV A and B genotypes and showed low cytotoxicity in HEp-2 and human primary bronchial epithelial cells (HBEC). Time-of-addition and pseudovirus assays demonstrated that lonafarnib inhibits RSV entry, but has farnesyltransferase-independent antiviral efficacy. Cryo-electron microscopy revealed that lonafarnib binds to a triple-symmetric pocket within the central cavity of the RSV F metastable pre-fusion conformation. Mutants at the RSV F sites interacting with lonafarnib showed resistance to lonafarnib but remained fully sensitive to the neutralizing monoclonal antibody palivizumab. Furthermore, lonafarnib dose-dependently reduced the replication of RSV in BALB/c mice. Collectively, lonafarnib could be a potential fusion inhibitor for RSV infection.

## Introduction

Respiratory syncytial virus (RSV) can cause severe bronchiolitis and pneumonia in infants and young children.^1,2^ Globally, ~33.1 million young children (<5 years old) are infected with RSV each year.^2^ RSV also causes respiratory disease in older and immunocompromised adults.^3^ Annually, there are up to 150,000 deaths worldwide from RSV infection, and most of them are in developing countries.^4^ RSV causes a substantial global disease burden, particularly in children and older adults.

So far, there are no approved small-molecule drugs for the treatment of RSV infection. Ribavirin, a broad-spectrum nucleoside analog, that targets RNA replication and transcription, has been approved for RSV therapy.^5^ No longer recommended due to its adverse side effects and efficacy.^6^ Palivizumab, a neutralizing monoclonal antibody, has been used in infants at highest risk for severe RSV disease.^7^ The European Medicines Agency (EMA) and the U.S. Food and Drug Administration (FDA) approved Nirsevimab for use in infants and young children only for the prevention of lower respiratory tract infections (LRTI) caused by RSV.^8,9^ Given the burden associated with RSV, there is an urgent need for the development of treatments.

RSV is an enveloped virus of the family *Pneumoviridae* virus with a negative-sense, single-stranded RNA genome, and targets ciliated bronchial epithelial cells in human airways.^10^ RSV entry into host cells consists of multistep processes that generally involve binding of the surface glycoproteins of the virion to the cellular receptors of human nucleolin and insulin-like growth factor 1 receptor.^11,12^ The fusion (F) glycoprotein is essential for entry and cell-to-cell fusion.^13–16^ RSV F is translated as an inactive precursor protein (F0), which is proteolytically processed by a host cell furin-like protease to generate three fragments (F1, F2, and pep27).^17^ A hydrophobic conserved domain at the N-terminus of the F1 subunit, termed the fusion peptide (FP), is considered to participate in the membrane fusion process.

The active F is a trimer of two disulfide-linked F2–F1 heterodimers expressed on the virus envelope and the surface of infected cells.^18,19^ RSV F merges virions and cell membranes by taking advantage of the difference in folding energy between two substantially different states [a metastable state (pre-fusion) and a stable state (post-fusion)].^20,21^ Therefore, inhibition of the two states exchanged during the fusion process may prevent RSV infection and could serve as a target for therapeutic intervention.

Small-molecule fusion inhibitors effectively suppress viral invasion. Currently, AK0529,^22^ JNJ-53718678,^23^ RV521,^24^ and GS-5806.^25^ are the most potent RSV fusion inhibitors in vitro and in vivo and are being clinically developed for RSV infection treatment. In particular, AK0529 has recently been shown to be efficacious against RSV infection in phase III efficacy trials.^26^ These inhibitors block pre-fusion to post-fusion conformational changes of F to interfere with RSV infection.

Lonafarnib is an oral active inhibitor that has been used to treat Hutchinson-Gilford progeria syndrome (HGPS) and hepatitis delta virus (HDV) infection by targeting farnesyltransferase.^27,28^ Using antiviral high-throughput screening, we discovered that lonafarnib is an inhibitor of RSV, and we characterized extensively the antiviral activity and mechanism of action of lonafarnib in vitro and in vivo. In addition, cryo-electron microscopy (cryo-EM) showed that lonafarnib binds to the pre-fusion F. The mutations in the F protein conferring reduced susceptibility to lonafarnib were identified using RSV F-mediated cell-to-cell fusion studies. Collectively, our data reveal that lonafarnib is an inhibitor of RSV infection that impairs the viral fusion process.

## Results

### The activity of lonafarnib against RSV infection

**Fig. 1:**
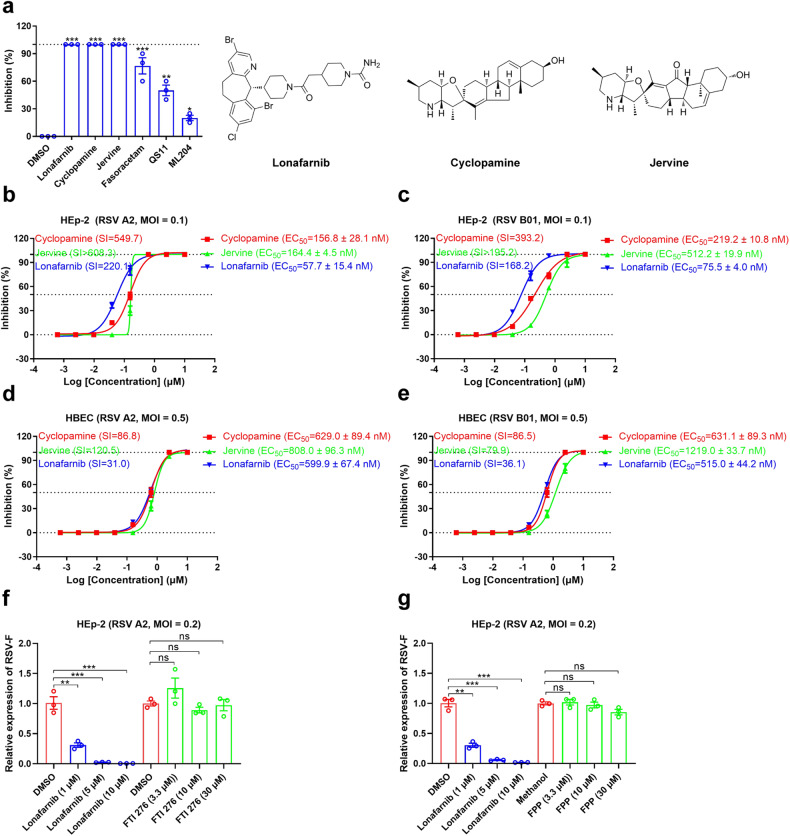
**Activity of lonafarnib against RSV infection. a** Antiviral activity of six compounds (lonafarnib, cyclopamine, jervine, fasoracetam, QS11, and ML204) from high-throughput screening at 5 μM against RSV infection at a multiplicity of infection (MOI) of 0.1 in a cytopathic effect (CPE) inhibition assay with HEp-2 cells. The chemical structures of lonafarnib, cyclopamine, and jervine with antiviral activity are in the top three. For statistical analysis, one-way ANOVA compared with the DMSO group was used. Antiviral activity of cyclopamine, jervine, and lonafarnib against RSV A2 (**b**) and B01 (**c**) at an MOI of 0.1 in a CPE inhibition assay with HEp-2 cells. Antiviral activity of cyclopamine, jervine, and lonafarnib against RSV A2 (**d**) and B01 (**e**) in a fluorescence focus assay (FFA) with HBEC. **b**–**e** Selectivity index (SI) = CC_50_/EC_50_ were calculated for all compounds tested. Treatment with the farnesyltransferase antagonist FTI 276 (**f**) and agonist FPP (**g**) did not affect RSV infection. FTI 276 and FPP were tested at concentrations exceeding their EC_50_. Lonafarnib (10, 5, 1 μM) was used as a control. For statistical analysis, non-parametric Mann-Whitney for comparison was used. The results are representatively shown with three random experiments. The error bars indicate mean ± SEM. *P < 0.05, **P < 0.01, ***P < 0.001; ns, not significant

To discover novel antiviral therapies against RSV, we performed cell-viability-based antiviral high-throughput screens in HEp-2 cells. Based on inhibition of the cytopathic effect (CPE) induced by RSV infection, 6 out of 2579 compounds of the in-house library could inhibit RSV infection at 5 μM (Fig. 1a and Supplementary Fig. 1a). Three of these six compounds (lonafarnib, cyclopamine and jervine) showed potent inhibitory activities on RSV infection (Fig. 1a). Cyclopamine and jervine, which are *Veratrum* steroidal alkaloids, have been previously reported to be potent inhibitors that block RSV infection.^29,30^ The results demonstrated that the method used is feasible. Further analysis indicated that the prenylation inhibitor lonafarnib, similar to cyclopamine and jervine, inhibits RSV A2 in a dose-dependent manner with 50% effective concentration (EC_50_) values of 57.7 ± 15.4 nM in HEp-2 cells (Fig. 1b). Lonafarnib also inhibits RSV B01 with EC_50_ of 75.5 ± 4.0 nM in HEp-2 cells (Fig. 1c). The selectivity index (SI) of lonafarnib exceeds 168.2 in HEp-2 cells (Fig. 1b, c and Supplementary Fig. 2a), indicating its safety at the cellular level. Furthermore, lonafarnib inhibits the RSV genotypes ON1 (subgroup A) and BA9 (subgroup B), which are now the dominant genotypes globally.^31,32^ (Supplementary Fig. 1b).

We further evaluated the antiviral activity of lonafarnib in the human bronchial epithelial cells (HBEC). Treatment with lonafarnib resulted in a dose-dependent reduction in RSV A2 replication with EC_50_ values of 599.9 ± 67.4 nM, showing similar potency to cyclopamine and jervine (Fig. 1d). Similarly, lonafarnib demonstrated robust antiviral efficacy against RSV B01 in HBEC with EC_50_ values of 515.0 ± 44.2 nM (Fig. 1e). Fifty percent cytotoxicity concentration (CC_50_) of lonafarnib in HBEC is 18.6 μM (Supplementary Fig. 2b). Together, lonafarnib effectively suppresses RSV infection.

Prenyltransferases are responsible for the covalent addition of isoprenoids to proteins.^33^ FTI 276 and lonafarnib, two specific inhibitors of farnesyltransferase, reduced total protein farnesylation whereas farnesyl pyrophosphate (FPP), an agonist of farnesyltransferase, caused a significant increase in farnesylation. FTI 276 and FPP had no effect on RSV infection in HEp-2 cells that were consistent with the reported findings.^34^ (Fig. 1f, g), but lonafarnib dose-dependently reduced RSV infection relative to untreated cells (Fig. 1f, g). These results demonstrate that lonafarnib inhibits infection of RSV, but has farnesyltransferase-independent antiviral efficacy in vitro.

### Lonafarnib inhibits the entry process of RSV

**Fig. 2:**
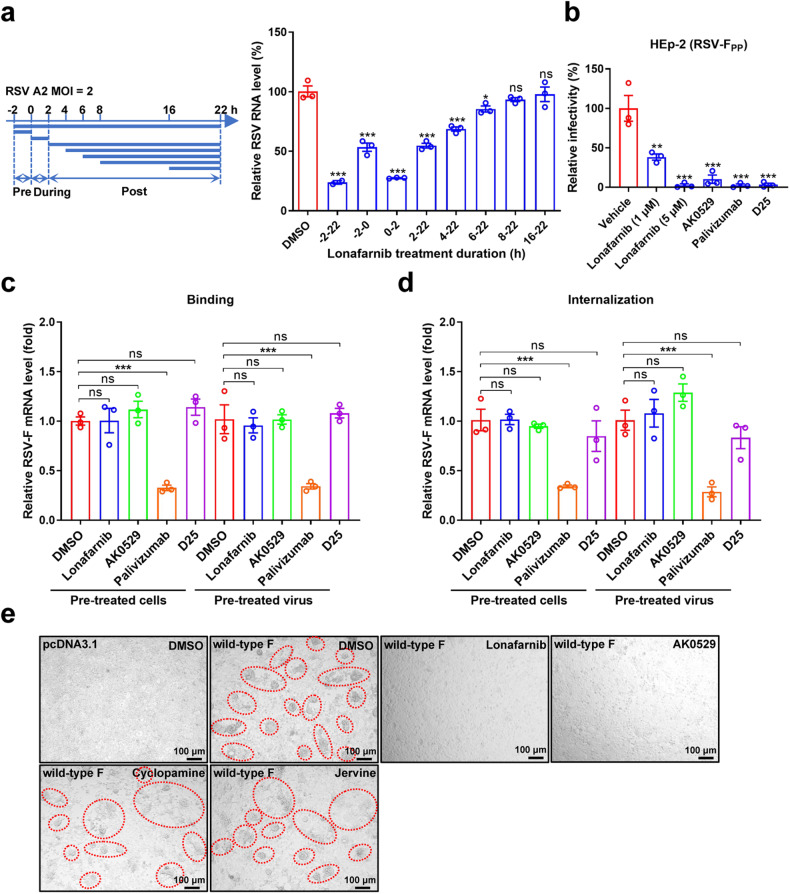
**Lonafarnib inhibits the entry process of RSV. a** Time-of-addition experiment of lonafarnib. Schematic illustration of the time-of-addition experiment (left). HEp-2 cells were infected with RSV A2 at an MOI of 2, and treated with 3.3 μM lonafarnib pre (−2–0 h), during (0–2 h), and post (2, 4, 6, 8, and 16 h) infection; 0.03% DMSO was added at the same time as in the control. The viral RNA was extracted and measured at 22 h postinfection (right). Statistical significance was assessed by Student’s *t*-test compared with the DMSO group. **b** HEp-2 cells were infected with the pseudotypes harboring RSV F protein (RSV-F_PP_) and treated with lonafarnib (1 or 5 μM), AK0529 (5 μM), palivizumab (10 μg/mL), and D25 (10 μg/mL). At 48 h postinfection, the luciferase activity of RSV-F_PP_ was analyzed. Lonafarnib did not modulate RSV binding and internalization. RSV A2 was incubated with HEp-2 cells pretreated with lonafarnib, AK0529, palivizumab, or D25 mAb (**c**, **d**, left), or RSV A2 was incubated with lonafarnib, AK0529, palivizumab or D25 mAb before infection (**c**, **d**, right). Cells were collected and the viral RNA was detected. **e** Lonafarnib inhibits the RSV F protein-induced cell-to-cell fusion process. RSV F protein was transiently expressed in HEK293T cells. Different compounds at 5 μM were diluted and added to the wild-type RSV-F-expressing cells. The cell-to-cell fusion was observed using microscopy on day 2 after compound addition. The results are representatively shown with three random experiments. The error bars indicate mean ± SEM. Statistical significance was assessed by ANOVA for comparison. *P < 0.05, **P < 0.01, ***P < 0.001; ns, not significant

To determine the stage of the viral life cycle blocked by lonafarnib, a qRT-PCR-based time-of-addition assay was performed. Our results showed that RSV infection reduced significantly when lonafarnib was added at full-time (−2–22 h) and entry (0–2 h). However, there was weak inhibition at the post-entry stages, suggesting it blocks RSV mainly through the suppression of virus entry (Fig. 2a). Next, we assessed the efficacy of lonafarnib in inhibiting entry using RSV pseudoparticles (RSVpp) assays. Our results demonstrated that lonafarnib inhibits the RSV entry process, consistent with the result from the time-of-addition assay (Fig. 2b).

Treatment of cells or virions with lonafarnib, AK0529, or D25 mAb neither affects the binding of the virus to cells (Fig. 2c) nor the internalization of the virus (Fig. 2d), while RSV F specific neutralizing antibodies (palivizumab) inhibit the binding and internalization of the virus. RSV F protein could induce directly membrane fusion and syncytium formation. Similar to the fusion inhibitor AK0529, lonafarnib significantly inhibited syncytium formation induced by overexpression of the RSV F protein (Fig. 2e, upper panel). Even in inhibiting RSV replication, jervine and cyclopamine did not affect F-induced syncytium formation (Fig. 2e, lower panel). These results strongly suggest that lonafarnib might inhibit the RSV fusion process.

### Cryo-EM structure of pre-fusion F in complex with lonafarnib

To further answer whether lonafarnib affects the membrane fusion of the virus by directly acting on F, we expressed the pre-fusion conformational RSV F protein (DS-Cav1).^20^ in HEK293F cells (Supplementary Fig. 3a). Surface plasmon resonance (SPR) experiment indicated that lonafarnib interacts with the F protein with a dissociation equilibrium constant (*K*_D_) of 20.1 μM, which is similar to AK0529 with the F protein with a *K*_D_ of 44.3 μM (Supplementary Fig. 4).

**Fig. 3:**
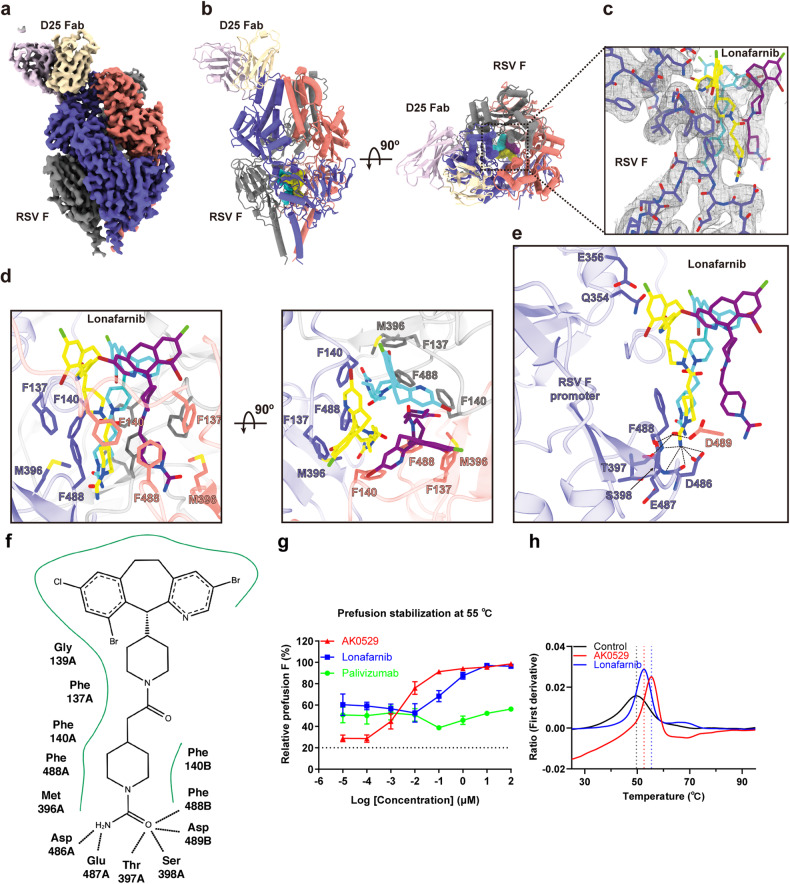
**Cryo-EM structure of pre-fusion F in complex with lonafarnib. a** Cryo-EM map of lonafarnib bound to RSV F-D25 Fab complex and colored according to the subunit. Slate blue, RSV F promoter **a**; Salmon, RSV F promoter **b**; Gray, RSV F promoter **c**; Wheat and thistle, D25 Fab; Lonafarnib, yellow, cyan, and purple. **b** Orthogonal views of lonafarnib bound to the RSV F-D25 Fab complex. **c** Density map and constructed model of lonafarnib near the lonafarnib pocket. The density map is shown as mesh and the threshold level is 0.06. **d** Close-up view of the hydrophobic interactions in the lonafarnib-binding site. **e** Close-up view of the hydrophilic interactions of one lonafarnib molecule. Hydrogen bonds are indicated with dashed lines. **f** 2D ligand-interaction diagram of lonafarnib binding to pre-fusion RSV F. **g** The relative percentage of RSV F remaining in the pre-fusion conformation after a 55 °C heat shock for 15 min was performed with an increasing concentration of AK0529, lonafarnib, or palivizumab. *n* = 3 biological replicates. **h** Differential scanning fluorimetry (DSF) for the stability of lonafarnib and AK0529 to DS-Cav1. The black, blue, and red lines represent the melting temperature (*T*_*m*_) value fitted curve for DS-Cav1 incubated with DMSO, AK0529, or lonafarnib, respectively. The results are representatively shown with three random experiments

To elucidate the molecular basis of fusion inhibition, the structure of lonafarnib bound to RSV F (DS-Cav1) was determined at a resolution of 3.17 Å with cryo-EM (Fig. 3a, b, Supplementary Figs. 3 and 5). Analysis of the cryo-EM density revealed the presence of lonafarnib within the central cavity of the pre-fusion F, positioned below the hydrophobic FP (Fig. 3c). Notably, our analysis indicated that three molecules of lonafarnib could be accommodated within this density. In the binding pocket, lonafarnib establishes hydrophobic interactions with Phe137, Phe140, Met396, and Phe488 (Fig. 3d, f). Furthermore, the charged appendages of lonafarnib extend into a negatively charged pocket, forming hydrogen bonds with residues Ser398, Asp486 and Asp489 as well as the main chain of Thr397, Glu487, and Phe488 (Fig. 3e, f). These observations strongly suggest that lonafarnib binds the FP domain to heptad repeat B (HRB), thereby stabilizing the pre-fusion conformation of the F protein.

The binding mode of lonafarnib suggested that it may stabilize the pre-fusion conformation of the F protein and prevent triggering the post-fusion state. To validate this hypothesis, we performed a stabilization of pre-fusion F assay that uses a pre-fusion-specific antibody (D25) to assess the conformation changes of RSV F.^19^ Lonafarnib was incubated with supernatants of HEK293T cells expressing RSV F, and they were then heat shocked at 55 °C, which triggered the conversion of RSV F from the pre- to post-fusion conformation.^35^ The addition of lonafarnib or AK0529 prevented the triggering of RSV F in a dose-dependent manner and had an inverse correlation between the amount of pre-F and the amount of post-F, but palivizumab did not (Fig. 3g). Similarly, the result of differential scanning fluorimetry (DSF) showed that lonafarnib and AK0529 also stabilized the conformation of the pre-fusion F protein (Fig. 3h). These findings confirmed that lonafarnib is an inhibitor that stabilizes the pre-fusion F protein and prevents its transition to the post-fusion conformation.

### HDX-MS solution structural analysis reveals that lonafarnib stabilizes pre-fusion F

**Fig. 4:**
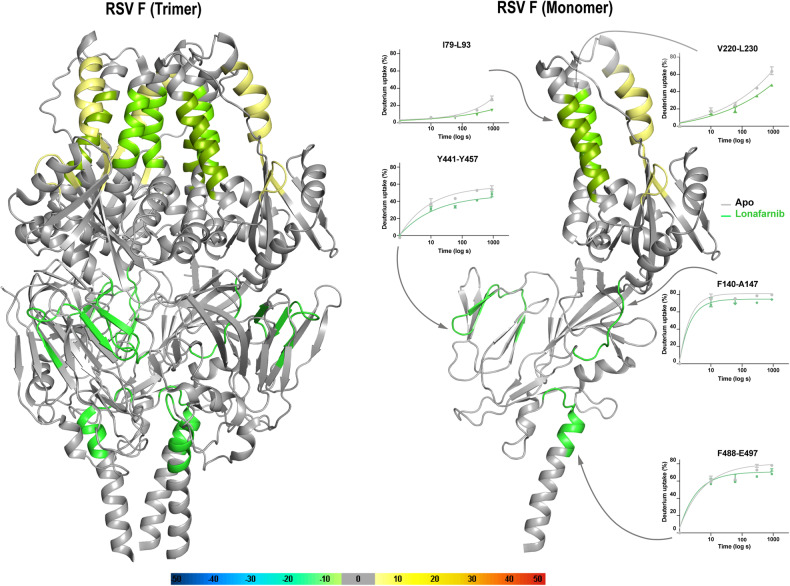
**HDX-MS analysis and structural mapping of lonafarnib binding to the fusion protein of RSV**. Differential HDX-MS analysis of RSV pre-fusion F trimer and monomer ± lonafarnib is shown as the change in deuterium uptake mapped onto the cryo-EM structure (PDB: 8KG5). Deuterium uptake plots for peptides affected by ligand binding in the absence (gray) or presence (green) of lonafarnib. The data represent mean ± SEM of three experimental replicates

The hydrogen/deuterium exchange coupled to MS (HDX-MS) was used to investigate the dynamic conformational change in RSV F protein binding lonafarnib. Five regions (79IKQELDKYKNAVTEL93, 140FLLGVGSA147, 220VIEFQQKNNRL230, 441YVSNKGVDTVSVGNTLY457, and 488FDASISQVNE497) show clear lower deuterium exchange rate, meaning decreased structural fluctuation and lower solvent accessibility located on the F1/F2 subunit, FP, and HRB (Fig. 4 and Supplementary Fig. 6). The regions 140FLLGVGSA147 and 488FDASISQVNE497 located on the same pocket surface that can accommodate the ligand and deuterium uptake plots indicate high solvent exchange (~80%) relative to other observed solvent-exposed peptides (Fig. 4). In line with this, lonafarnib formed several H-bonds with the critical residue 486DEFD489 and hydrophobic interactions with the residues Phe137 and Phe140 according to the solved cryo-EM structure. In addition, we noticed two regions (190FKVLDLKN197 and 198YIDKQLLPIL207) located on the heptad repeat A (HRA) show destabilization with higher solvent accessibility, which varies from HRB and is unfavorable for RSV F post-fusion formation (Fig. 4 and Supplementary Fig. 6).^36^ These data further support that RSV F is the bona fide target of lonafarnib, which binds and stabilizes pre-fusion F to inhibit RSV infection.

### Mutations in the F protein reduce sensitivity to lonafarnib

**Fig. 5:**
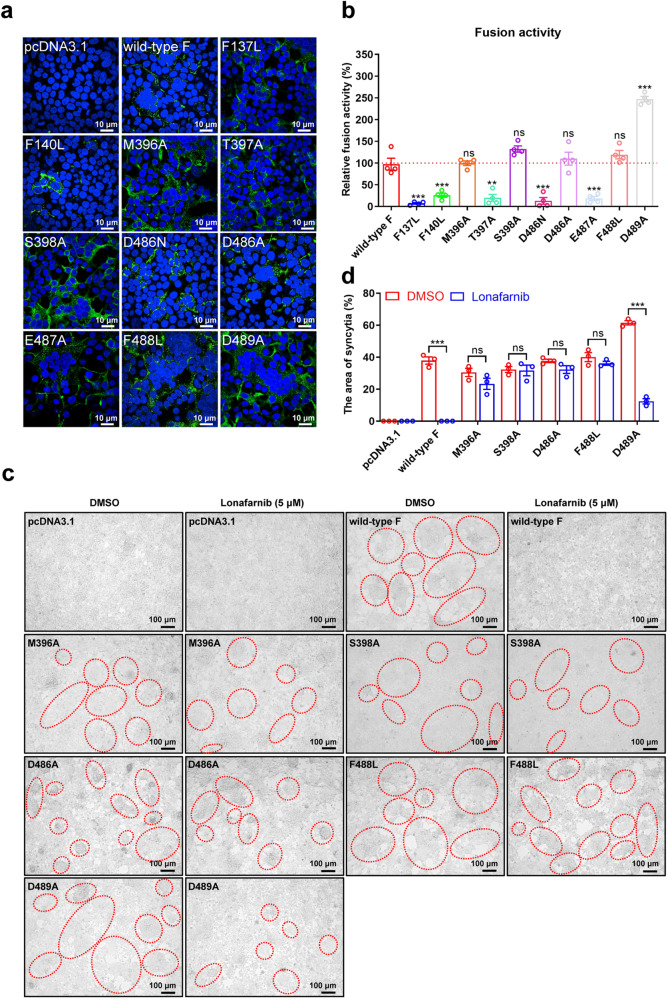
**Impact of RSV F mutations on cell-to-cell fusion activity and lonafarnib binding. a**, HEK293T cells were transfected with wild-type F, F137L, F140L, M396A, T397A, S398A, D486N, D486A, E487A, F488L, or D489A plasmids for 36 h. The cells were fixed followed by staining, and imaging using confocal microscopy. The pcDNA3.1 plasmid was used as a vector control. **b** Relative fusion activity for RSV F mutations (such as F137L, F140L, M396A, T397A, S398A, D486N, D486A, E487A, F488L, or D489A) was normalized to wild-type F. Statistical significance was assessed by ANOVA for comparison. **c** RSV F constructs carrying M396A, S398A, D486A, F488L, or D489A mutations were tested for their ability to induce the cell-to-cell fusion process during lonafarnib (5 μM) addition to the cells. The pcDNA3.1 plasmid was used as a vector control. **d** Quantification of the area of the syncytia was analyzed using Image J. For statistical analysis, non-parametric Mann-Whitney for comparison was used. The results are representatively shown with three random experiments. The error bars indicate mean ± SEM. **P < 0.01, ***P < 0.001; ns, not significant

**Fig. 6:**
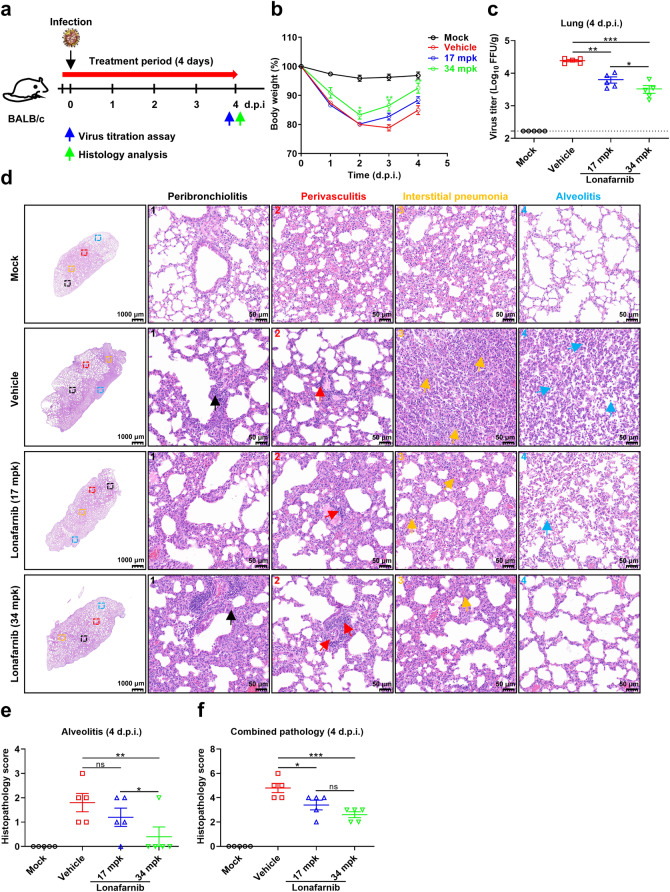
**In vivo efficacy of lonafarnib against RSV infection in BALB/c mice. a–f**, BALB/c mice were orally administered 17 or 34 mpk lonafarnib for the treatment group (*n* = 5 mice/group) or vehicle solution only for the control group (*n* = 5 mice/group) twice daily for 4 days. At 2 h after lonafarnib treatment, the mice were intranasally inoculated with RSV A2 (1 × 10^6^ PFU). The mock and vehicle groups were used as controls. **a** Schematic of BALB/c mice infection and treatment. **b** Mouse body weights were monitored for up to 4 days postinfection. **c** Mice infected with RSV A2 were killed on day 4 postinfection for the detection of infectious viral titers in the lung tissue using FFA (*n* = 5). **d** Histological analysis visualizing the virus-induced pathology in the lung of mice infected with RSV A2 on day 4 postinfection. Four parameters of pulmonary inflammation were evaluated: i, peribronchiolitis (black arrows: inflammatory cell infiltration around the bronchioles); ii, perivasculitis (red arrows: inflammatory cell infiltration around the small blood vessels); iii, interstitial pneumonia (yellow arrows: inflammatory cell infiltration and thickening of alveolar walls); iv, alveolitis (blue arrows: cells within the alveolar spaces). The results are representatively shown from a mean score of combined pathology. Representative images of the lungs are indicated by black squares with numbers, and enlarged in images 1, 2, 3, and 4. Scale bar, 1,000 µm, and 50 µm for enlarged images 1–4. Quantitative scoring of the pathology of the lung from mice on day 4 postinfection. The blinded scorings of the alveolitis (**e**) and combined pathology (**f**) were shown, respectively. Histopathology scores for each pulmonary inflammation on a scale of 0–4, where 0 is a normal healthy lung and 4 is severe confluent areas of pathology. The data are representative of at least two experiments. The error bars are mean ± SEM. Statistical differences were determined by two-way ANOVA in **b**, **c**, **e** and **f**. *P < 0.05, **P < 0.01, ***P < 0.001; ns, not significant

To further verify the function of the amino acid binding sites of F interacting with lonafarnib, we constructed a series of expression plasmids of the F protein with amino acid mutations. We mutated Phe (F) to Leu (L) (F137L, F140L, and F488L), and Asp (D) to Asn (N) (D486N), while the other amino acids mutated to Ala (A) (M396A, T397A, S398A, D486A, and D489A). Confocal microscopy showed all the F mutants retain cell membrane localization in HEK293T cells (Fig. 5a). The F137L, F140L, T397A, and E487A mutants lost their ability to induce syncytium, indicating that Phe137 and Phe140 in the fusion peptide domain, Thr397 in main chain, and Glu487 in HRB play essential roles in F-mediated fusion (Fig. 5b and Supplementary Fig. 7). As previously reported,^21^ expression of the D486N mutation resulted in low levels of cell-to-cell fusion activity, ~4-fold below than that of wild-type F (Fig. 6b). The M396A, S398A, D486A, and F488L mutants have the same ability to induce fusion as the wild-type F protein; however, lonafarnib did not or little inhibit syncytia formation induced by M396A, S398A, D486A, and F488L (Fig. 5c), indicating these mutations might lead to the resistance of RSV to lonafarnib. Compared with wild-type F, lonafarnib did not completely inhibit the D489A mutant-induced cell-to-cell fusion activity, which is ~2.5-fold above that of the wild-type F (Fig. 5b, c). These results indicate the M396A, S398A, D486A, F488L, and D489A mutations of RSV F might have led to the resistance of RSV to lonafarnib.

### In vivo efficacy of lonafarnib against RSV infection in BALB/c mice

Finally, we evaluated the in vivo antiviral efficacy of lonafarnib against RSV in a BALB/c mouse model. Two hours before the 8-week-old BALB/c mice were intranasally inoculated with 1 × 10^6^ PFU RSV A2, the mice were orally administered 17 or 34 milligrams per kilogram (mpk) lonafarnib for the treatment group or vehicle solution only for the control group twice daily for 4 consecutive days (Fig. 6a). In the RSV-infected BALB/c mice, 34 mpk lonafarnib improved the body weight of the mice from day 1 postinfection compared with that of the vehicle group in the prophylactic group (Fig. 6b). The viral titers in the lung of the lonafarnib-treated mice were also reduced, as evidenced by the 3.8- (*P* < 0.01) and 7.4-fold (*P* < 0.001) decrease in the treatment group of 17 mpk and 34 mpk, respectively (Fig. 6c). Administered two hours after RSV infection (Supplementary Fig. 8a), 17 mpk and 34 mpk of lonafarnib also reduced pulmonary viral titers 3.7- and 4.1-fold (Supplementary Fig. 8c), while mild improvement of the body weight was observed only at 4 days post-infection (Supplementary Fig. 8b).

Next, we performed hematoxylin-eosin (H&E) staining to appraise virus-induced lung tissue damage. The vehicle-treated mice showed severe inflammatory infiltrations in the bronchioles, small blood vessels, alveoli septa, and alveoli on day 4 postinfection (Fig. 6d and Supplementary Fig. 8d). In contrast, a mild lung infiltration was observed in the prophylactic lonafarnib-treated mice, demonstrating that lonafarnib treatment can reduce lung injury after RSV infection (Fig. 6d). For therapeutic treatment, only 34 mpk lonafarnib reduced lung damage on day 4 postinfection compared with the vehicle group (Supplementary Fig. 8d). Quantitative pathology scores showed the same trends with the H&E pathology observation (Fig. 6e, f and Supplementary Fig. 8e, f). Overall, the in vivo efficacy study provided evidence for the potential prophylactic and therapeutic treatment usage of lonafarnib against RSV infection.

## Discussion

RSV causes a large burden of serious respiratory diseases worldwide.^1,2,37^ There are currently no approved RSV-specific therapeutic small molecules drugs available. In this study, we found that a farnesyltransferase inhibitor lonafarnib effectively suppressed the infection by RSV. Lonafarnib inhibits different RSV genotype infections in culture cell and primary cell HBEC, as well as reduced the RSV infection in a mouse model. Mechanically, lonafarnib targets the F protein instead of farnesyltransferase to inhibit the entry of RSV. Structural analysis showed that lonafarnib directly binds to the trimer of F and inhibits pre- to post-fusion conformational changes, thereby inhibiting F-mediated fusion. The repurposing of approved lonafarnib provides an alternative approach to the treatment of RSV infection.

Farnesyltransferase catalyzes the transfer of a farnesyl moiety from farnesyl pyrophosphate to protein. Prenylation, a post-translational modification, leads to farnesylation or geranylgeranylation, enhancing the hydrophobicity of proteins, which is crucial for their effective insertion into plasma and/or organelle membranes. Prenylated proteins function in several signaling pathways that are responsible for basic cell operations.^38,39^ An imbalance in protein prenylation modification leads to various diseases such as progeria and cancer.^40–42^ Farnesyltransferase is an important drug target, and its specific inhibitors and agonists have shown good prospects.^40,42,43^ The farnesyltransferase inhibitor lonafarnib has been used to treat HSPG.^27^ Lonafarnib inhibits HDV infection by blocking the large hepatitis D antigen prenylation by the cellular farnesyltransferase at a conserved Cys-residue (Cys-211) within the C-terminal extension.^28,43^ In this study, lonafarnib inhibited RSV infection using a completely different mechanism. Lonafarnib targets the F protein instead of cellular farnesyltransferase. Thus, the other farnesyltransferase inhibitor (FTI 276) or agonists (FPP) cannot inhibit RSV infection.

The F protein is thought to be essential for RSV entry and cell-to-cell fusion,^13–16^ which exists in two distinct forms (pre-fusion and post-fusion). The pre-fusion F, which is embedded in the viral membrane,^19^ is the predominant form found on the surface of virions using electron cryotomography.^44^ It triggers conformational changes in the pre-fusion F protein that expose the buried hydrophobic FP when RSV contacts cell surfaces such as attachment proteins and cell surface receptors. These interact with FP of neighboring molecules to form rosette-like structures to merge virus and cell membranes.^20,21^ During the RSV fusion process, the FP and HRB undergo significant conformational rearrangements.^21^ The cryo-EM structure showed that three lonafarnib molecules can bind simultaneously to an F trimer with a third axis of symmetry. This suggests a new anti-RSV drug design and improvement strategy, such as combining three molecules. Lonafarnib interacts with 10 sites at the F protein; Phe137 and Phe140 are part of the RSV F fusion peptide, while Asp486, Glu487, Phe488, and Asp489 are in the HRB (Supplementary Fig. 7). The binding site-based mutations (M396A, S398A, D486A, F488L, and D489A) showed good fusion ability, but confered lonafarnib resistance to the fusion activity of RSV F. This mode of binding is conservative for RSV fusion inhibitors, including JNJ-2408068,^21^ AK0529,^45^ RV521.^46^ and TMC-353121,^47^ which occupy all three lobes of the binding pocket. As with AK0529.^45^ and TMC-353121,^48^ escape selection experiments have shown that continued presence of lonafarnib can lead to RSV resistance (Supplementary Fig. 9). The antiviral activity and binding mode of lonafarnib provide a guide for improving inhibitory activity.

Lonafarnib binds inside the central cavity of the pre-fusion F protein and interacts with the FPs, preventing their insertion into host cell membranes. Monoclonal antibody (mAb) D25 binds a flexible region of the antigenic site Ø on either monomeric or trimeric pre-fusion F.^19^ Analysis of the pre-fusion-specific mAb D25 indicated that lonafarnib inhibits heat-induced conformational changes in the F protein (Fig. 3g), which was further confirmed using DSF assay (Fig. 3h). In addition, the HDX-MS analysis of pre-fusion F in complex with lonafarnib indicated that lonafarnib stabilizes the pre-fusion F state but not the post-fusion form. Consistent with our findings, the fusion inhibitors (i.e., JNJ-49153390, TMC-353121, GPAR-3710, and BMS-433771) stabilize pre-fusion F conformation and hinder its conversion to the post-fusion state post-fusion.^21,49^

Virus membrane proteins are important targets for antiviral therapy.^50–52^ The neutralizing mAbs, such as RSV (palivizumab^53^ and nirsevimab,^54^) SARS-CoV-2 (amubarvimab/romlusevimab,^55^) and Influenza A (MEDI8852,^56^) effectively block virus infection and has been widely used. The biggest challenge to the use of neutralizing mAbs is the variation of virus surface proteins. Investigational RSV small molecule fusion inhibitors were designed to block virus entry into host cells. Currently, there are at least four antiviral agents being tested in clinical trials (i.e., AK0529,^22^ JNJ-53718678,^23^ RV521,^24^ and GS-5806.^25^) AK0529^45^ and RV521.^46^ showed a good inhibitory effect in vivo. It reported that 1 log_10_ and 1.9 log_10_ of viral titer reduction in the lung of infected mice were achieved at the dose of 12.5 mpk and 50 mpk AK0529 compared to the vehicle group,^45^ respectively. Compared to the control group, virus titers in the lungs of mice treated with 1, 10, and 50 mpk RV521 were reduced by 0.7 log_10_, 1.1 log_10_, and 1.6 log_10_, respectively.^46^ Compared with AK0529 and RV521, prophylactic lonafarnib treatment presented a comparable viral inhibitory effect with a 0.9 log_10_ reduction of viral titer in the lung at 34 mpk. However, therapeutic efficacy of lonafarnib was lower compared with above-mentioned clinical-stage inhibitors. It is possible to further improve the antiviral effect by increasing the dosage or changing the mode of administration. The cotton rats are more permissive for RSV than BALB/c mice,^57,58^ the antiviral efficacy of lonafarnib in cotton rats is anticipated to limit RSV infection. Given that lonafarnib also inhibits farnesyltransferase activity and may therefore have unwanted side effects when administered orally at high doses. It is possible that combination therapy with lonafarnib and drugs that inhibit other viral activities, such as for instance the viral nucleoprotein,^59^ improve the efficacy/side effect ratio. Lonafarnib and the reported several fusion inhibitors have shown excellent effects targeting the fusion process. In consideration of the conservation of the fusion process and the protective effects on different RSV variants, such as genotypes B01, ON1, and BA9, lonafarnib has the potential to function as an inhibitor of different RSV genotypes.

Small molecule fusion inhibitors have been developed, targeting the RSV F protein and effectively interfering with the membrane fusion process, yet their druggability remains largely unknown. Our data provided evidence for the potential of the repurposing of lonafarnib against RSV infection, particularly in patients with existing HGPS, HDV, or cancer comorbidities.

## Materials and Methods

### Cells and viruses

HEp-2 cells, HEK293T cells, and primary HBEC used in this study were cultured in a humidified incubator under 37 °C with 5% CO_2_. HEp-2 and HEK293T cells obtained from the ATCC were grown in DMEM (Hyclone), supplemented with 10% fetal bovine serum (FBS, Gibco) and 1% penicillin–streptomycin (Life Technologies). HBEC were purchased from Procell (CP-H009) and cultured in the CM-H009 medium. HEK293F cells obtained from the ATCC were maintained in SMM 293-TII expression medium (M293TII, Sino Biological) and incubated in an orbital incubator shaker at 37 °C with 5% CO_2_.

RSV A2 (GenBank: KT992094) and RSV B01 (GenBank: AF013254) strains were propagated in HEp-2 cells. Briefly, 3 days postinfection, the supernatant was collected and the viral titer was determined in HEp-2 cells using TCID_50_ assay. pSMART-BAC vector containing a full-length cDNA copy of the RSV-A-0594 (ON1 genotype) antigenome and enhanced green fluorescent protein (GFP) together with helper plasmids (pCG vector) expressing the RSV-A-0594 N, P, M2-1, and L proteins, kindly provided by Professor Martin Ludlow.^60^ For rescue of recombinant RSV, 5 × 10^5^ HEp-2 cells were infected with MVA-T7 (MOI = 2) for 1 h at 37 °C. Cells were then transfected with pSMART-BAC (1.6 μg) together with helper plasmids N (1.6 μg), P (1.2 μg), M2-1 (0.8 μg), and L (0.4 μg). Thereafter, cells were monitored for spreading foci of fluorescent cells The supernatant containing rRSV-A-0594-GFP virus (RSV ON1-GFP) was harvested at 5 to 6 days posttransfection. The RSV ON1-GFP virus was propagated as above described.

### High-throughput antiviral screening

The RSV A2-based high-throughput antiviral screening was performed in HEp-2 cells (1.3 × 10^4^) with a 96-well plate format. The in-house compound library was diluted to 5 μM with DMEM-10% FBS as the working concentration. Then the compounds were added to the indicated wells followed by infection with RSV A2 (MOI = 0.1). At 72 h post infection (hpi), the number of CPE-positive cells was read.

### Cytotoxicity assay

HEp-2 cells and HBEC (1.3 × 10^4^) were seeded in 96-well plates for 20 h. Compounds in a 4-fold dilution series were added to the cells and three wells were performed in parallel. After 72 h, the cells were incubated DMEM-10% FBS with 10% CCK8 reagent (Beyotime Biotechnology) for 30 min at 37 °C. The absorbance at 450 nm and 600 nm was read using the PerkinElmer Ensight reader.

### Evaluation of the in vitro antiviral activity

HEp-2 cells (1.3 × 10^4^) were seeded in 96-well plates for 20 h. The cells were infected with RSV (MOI = 0.1) and were incubated with cyclopamine, jervine, or lonafarnib. The antiviral activity of the compounds at 72 hpi was expressed as EC_50,_ the concentration of drug required to reduce the CPE by 50%.

HBEC (1.3 × 10^4^) were seeded in 96-well plates., were infected with RSV (MOI = 0.5), and were incubated with cyclopamine, jervine, or lonafarnib. At 72 hpi, the cells were fixed in 4% paraformaldehyde (PFA) for 15 min and then permeabilized with 0.3% Triton X-100 for 15 min at room temperature (RT). RSV was probed using anti-RSV-FITC (GeneTex, GTX36375) diluted 1:100, over a 1 h incubation at RT. Fluorescent images were acquired with an Evos M5000 Cell Imaging System (Thermo Fisher Scientific) at 4 × magnification. Fluorescence dots were quantified and normalized to the total nuclear count using Image J.

### Pseudotyped RSV production and infection

Lentivirus-based pseudotypes bearing the RSV fusion glycoprotein were generated by transfecting HEK293T cells.^61^ Briefly, 5 × 10^5^ cells were seeded into 6-well plates 20 h before transfection with 1.2 μg RSV F plasmids, 0.4 μg psPAX2 and 0.4 μg pWPXLd-Firefly-Luc transducing vector. The supernatants were collected and used to infect HEp-2 cells with lonafarnib, AK0529, palivizumab, or D25 treatment, or vehicle. The luciferase activity was evaluated at 48 hpi.

### Time-of-drug addition assay

HEp-2 cells were infected with RSV (MOI = 2) for 2 h at 37 °C. Lonafarnib (3.3 μM) was added to the infected cells at the following time points: preinfection (–2–0 h), during infection (0 – 2 h), and postinfection (2, 4, 6, 8, and 16 h). DMSO (0.03%) as a control. The intracellular RNA was harvested at 22 hpi and the viral RNA level was determined using qRT-PCR. The primer (F/R, 5’-3’) targets F gene: CGAGCCAGAAGAGAACTACCA/CCTTCTAGGTGCAGGACCTTA; *β-actin*: CTCGACACCAGGGCGTTATG/CCACTCCATGCTCGATAGGAT. The inhibition rates were calculated as the percentage of the viral RNA level relative to the control.

### Virus binding and internalization assay

HEp-2 cells (1.5 × 10^5^) were seeded on 12-well plates for 20 h for the binding assay. Cells or viruses were mixed with lonafarnib (5 μM) followed by infection with RSV (MOI of 10) and incubated on ice for 1 h. AK0529 (5 μM), palivizumab (20 μg/mL), and D25 mAb (20 μg/mL) were used as a control. The cells were lysed in Trizol for RNA extraction after five cycles of washing. For the internalization assay, after five cycles of washing, the cells were added to the medium supplemented with the drugs and then shifted into a 37 °C incubator for 1 h. Thereafter, freeze cells on ice and then treated with proteinase K (500 ng/mL) on ice for 1 h. After five additional washes, the cells were collected for RNA extraction. Afterward, the viral RNA level was determined using qRT-PCR.

### Virus-free syncytia assay

HEK293T cells (2 × 10^5^) were seeded in 12-well plates for 20 h, and transfected with with the codon-optimized RSV A2 F plasmid followed by replacement with drug-containing media after 6 h. The cell-cell fusion was observed 48 h after drug addition using microscopy. Cell-cell fusion activity was quantified using a luciferase expression system.^62^ HEK293T cells (2 × 10^5^, target cells) were seeded in 12-well plates for 20 h, and transfected with 1 µg of wild-type F, F137L, F140L, M396A, T397A, S398A, D486N, D486A, E487A, F488L, or D489A plasmids and 1 µg of pBD-NFκB (Hunan Fenghui Biotechnology), a plasmid expressing Gal4-NFκB. A second set of HEK293T cells (effector cells) were grown in 12-well plates and were transfected with 1 µg of pFR-Luc (Hunan Fenghui Biotechnology). At 12 h posttransfection, the cells were released and resuspended, and the effector cells (2.5 × 10^4^) were mixed with an equal amount of the target cells in a 96-well plate and incubated for an additional 24 h before measuring the luciferase activity using the One-LiteTM Luciferase Assay System (Vazyme Biotech Co. Ltd.) and the luminescence was read using the PerkinElmer Ensight reader.

### Cloning, expression, and purification of protein

The gene encoding the stabilized pre-fusion RSV F trimer (DS-Cav1) was synthesized (GenScript) and cloned into the pcDNA3.1 expression vector sequentially fused with T4-fibritin trimerization domain, prescission protease, Strep-Tag II and 6×His tag at the C-terminal.^20,63,64^ The recombinant plasmid was transfected into HEK293F cells with polyethylenimine (PEI). The cell supernatant was harvested, centrifuged, and filtered at 5 days post-transfection. The complex was initially purified with Ni^2+^-NTA resin (Cytiva) using an elution buffer (1×PBS pH 7.4, 500 mM imidazole). DS-Cav1 was further purified using a Superose 6 10/300 GL gel filtration column (Cytiva) with running buffer consisting of 1 × PBS pH 7.4, then concentrated to about 1 mg/mL.

### SPR assay

The interaction between DS-Cav1 and inhibitors was monitored using surface plasmon resonance (SPR) and a Biacore 8K (GE Healthcare) carried out at 25 °C in a multi-cycle mode. DS-Cav1 was immobilized on a Series S Sensor chip CM5 (Cytiva) to 4550.8 response units. Inhibitors with concentrations of 3.125, 6.25, 12.5, 25, and 50 μM were in buffer containing 10 mM PBS pH 7.4, 0.05% Tween 20, and 5% DMSO when testing interactions with DS-Cav1. The equilibrium dissociation constants (binding affinity, *K*_D_) for each pair of interactions were calculated using the Biacore^®^ 8 K evaluation software (Cytiva).

### HDX-MS assay

A completely automated system similar to that previously described was used to perform solution-phase amide HDX experiments, with slight modifications.^65^ 20 μL of D_2_O-containing PBS buffer was mixed with 5 μL of 10 μM RSV pre-fusion F, either with or without 10:1 molar equivalency of lonafarnib. The mixture was then incubated at 4 °C for a variety of time intervals (0, 10, 30, 60, and 900 s). Following exchange, unwanted forward or back exchange was minimized and followed by the protein denaturation with a quench solution (5 M urea, 50 mM TCEP, and 1% v/v TFA) at a 1:1 ratio to the protein. The resulting peptides were trapped on a C18 trap column (Hypersil Gold, Thermo Fisher) by flowing the samples through an in-house prepared immobilized pepsin column.^66^ at 50 μL min^−1^ (0.1% v/v TFA, 15 °C). The bound peptides were then gradient-eluted across a 1 mm × 50 mm C18 column (Hypersil Gold, Thermo Fisher) for 5 min at 4 °C (5–50% CH3CN w/v and 0.3% w/v formic acid). A high-resolution Orbitrap mass spectrometer (Fusion, Thermo Fisher) was used to directly analyze the eluted peptides. The experiment was performed in triplicate. To identify peptides, MS/MS experiments were performed using a Fusion Orbitrap mass spectrometer. The MS/MS *.mgf files converted from *.raw data files were submitted to Mascot (Matrix Science) for peptide identification. Peptides with a Mascot score of 20 or greater were incorporated into the peptide set used for HDX detection. To rule out the false positives, the MS/MS Mascot search was also performed against a decoy (reverse) sequence. The intensity-weighted average m/z value (centroid) of each peptide isotopic envelope was calculated with HDX Workbench.^67^

### Electron microscopy sample preparation and imaging

A quantity of 10 μL of the concentrated DS-Cav1 protein (1 mg/mL) was combined with an equal volume of D25 Fab fragments (1 mg/mL) in a PBS buffer solution. This mixture was then treated with Lonafarnib at a final concentration of 100 μM and allowed to incubate for a duration of 30 minutes on ice. Subsequently, 2.5 μL of this prepared sample was applied to a 300-mesh Quantifoil Cu 1.2/1.3 grid, which had been pre-treated with H_2_/O_2_ glow discharge process (Quantifoil, Micro Tools GmbH). The grid was further processed using a Thermo Fisher Vitrobot for blotting and was rapidly plunged into liquid ethane for freezing. Imaging was collected using a Thermo Fisher Krios G4 electron microscope (Thermo Fisher), which is outfitted with a cold field-emission source, a Selectris X energy filter, and a Falcon 4 detector. The energy filter was set to a slit width of 10 e^-^V to eliminate electrons that have undergone inelastic scattering. The EPU software was utilized to acquire image stacks at a resolution of 0.73 Å per pixel under an exposure dose of 50 e^-^/Å^2^.

### Image processing and 3D reconstruction

The dataset, consisting of 7819 image stacks, underwent correction for motion induced by the electron beam and estimation of the contrast transfer function (CTF) using the cryoSPARC software suite.^68^ A total of 725,628 particles were automatically identified and extracted using a 384-pixel box size. Following iterative 2D and 3D classification steps, a subset of 104,617 particles was selected for ab-initio reconstruction and heterogeneous refinement processes. Subsequently, a refined candidate model, along with 37,059 particles, was subjected to further refinement using NU-Refinement to produce the final cryo-EM map at a resolution of 3.17 Å. Additionally, the local resolution of the map was assessed using cryoSPARC, applying the gold-standard Fourier shell correlation (FSC) with a threshold of 0.143. All dataset processing is shown in Supplementary Fig. 5.

### Model building and refinement

The atomic model of lonafarnib bound to RSV F-D25 Fab complex was built based on the D25-RSV F crystal structure (PDB: 4JHW). To start, the D25-RSV F crystal structure was docked into the EM density map using Chimera. This was then followed by iterative manual fitting adjustment in Coot.^69^ and real space refinement in PHENIX.^70^ Ligands were placed into the EM density map using Coot. All figures were drawn using UCSF Chimera,^71^ UCSF ChimeraX,^72^ and PyMOL.^73^ The data collection and refinement statistics are presented in Supplementary Table. 1.

### Immunofluorescent assay

Immunofluorescent assay was performed as described previously.^74^ In brief, the samples were fixed with 4% PFA and then permeabilized in 0.3% Triton X-100, blocked in 5% BSA (Macklin, B824162) in PBS for 1 h and incubated with the mouse monoclonal antibodies 2F7 (Abcam, ab43812) at 4 °C overnight. Cells were then stained with donkey anti-mouse IgG (H + L) antibody, Alexa fluor 488 conjugated (Thermo Fisher, A-21202). After washing and counterstaining with DAPI, the samples were observed with a Perkin Elmer UltraView Vox confocal microscope.

### Enzyme-linked immunosorbent assay (ELISA)

RSV F glycoprotein constructs were derived from the A2 strain (accession no. P03420).^63,75^ The codon-optimized RSV F (1-513) with a C-terminal T4 fibritin trimerization motif, thrombin site, 6×His-tag, and Strep II tag was synthesized and subcloned into pcDNA3.1 vector. HEK293T cells were transfected with plasmids expressing RSV F. 48 h posttransfection, cell supernatants were collected, and detected the amount of total F with motavizumab (MCE, 677010-34-3). The equal supernatant containing RSV F protein (1 mg/mL) was heat-shocked at 55 °C for 15 min with an increasing concentration of AK0529, lonafarnib, or palivizumab. After heat shock, samples were transferred to an ELISA plate coated with D25 mAbs, incubated at 37 °C for 2 hours, and then HRP conjugated 6×His, His tag monoclonal antibody (Proteintech, HRP-66005) was added for detection. The absorbance at 450 nm was quantified with the plate reader. The remaining pre-fusion F in the supernatant was determined with the DMSO group as a control and plotted using GraphPad Prism 8.0.

### Differential scanning fluorimetry

The DSF was used to follow the thermal unfolding event of DS-Cav1 with a Prometheus Panta (NanoTemper Technologies). The fluorescence was recorded at 330 and 350 nm over a temperature gradient scan. The temperature at the transition point of the fluorescence ratio 330–350 nm corresponds to the melting temperature (*T*_*m*_). The shift in *T*_*m*_ in the presence of a ligand is interpreted as potential binding. DS-Cav1 was diluted to 0.25 mg/mL with assay buffer (1 × PBS, pH 7.4). Inhibitors were dissolved in DMSO to a final concentration of 2 mM. Approximately 20 μL of DS-Cav1 at 0.25 mg/mL was mixed with 1 μL of 2 mM inhibitor. The samples were incubated for 10 min at 25 °C before loading them with high-sensitivity capillaries into the Panta. The excitation power was set between 100%, and the tested temperature range was from 25 °C to 95 °C.

### In vivo efficacy of lonafarnib against RSV in mice

Forty female BALB/c mice at the age of 8 weeks were purchased from Human SJA Laboratory Animal. (Changsha, China). The mice were housed in a specific pathogen-free environment under standard conditions. Lonafarnib was administered orally to the mice 2 h before infection or 2 h post-infection in the prophylactic group and therapeutic group, respectively. For the drug treatment group, mice were orally administered 17 or 34 mg kg^−1^ dose^−1^ (mpk) lonafarnib diluted in 200 μL 5% DMSO/20% hydroxypropyl-beta-cyclodextrin twice daily (BID) for 4 days. The mice were anesthetized with isoflurane and inoculated intranasally with 1 × 10^6^ PFU of RSV A2 on the day of infection. The weight of the mice was recorded daily. The mice were killed on day 4 postinfection, and the lung tissues was sampled for virological and histopathological analyses. After weighing the right lung tissue, 1000 μL PBS was added into the tube, grounded with a grinding instrument, and 100 μL of the grinding tissue was used to determine the virus titer by FFA. In short, the cells were infected with 100 μL supernatants of the grinding tissue or 4-fold gradient dilution by centrifugation (350 *g* × 30 min) at 30 °C. At 24 hpi, cells were fixed in 4% PFA and then permeabilized with 0.3% Triton X-100. RSV was probed using anti-RSV-FITC (Cat No. GTX36375) diluted 1:100 in PBS, overnight incubation at 4 °C. Fluorescence dots were quantified by Image J. The virus titer is calculated according to the following formula: virus titer (FFU/g) = average number of fluorescent foci per well × dilution degree × volume index × volume × lung tissue weight index (converted to g). The left lung tissue was fixed using 4% formaldehyde immersion fixation, embedded, sectionized, and stained with H&E to observe the pathological changes. Slides were scored blindly by two independent pathologists on a 0–4 severity scale.^76^

### In vitro selection for RSV resistance to lonafarnib

To select for the development of drug resistance against lonafarnib, RSV ON1-GFP reporter virus (rRSV-A-0594, GenBank: MW582528) was cultured in the presence of increasing concentrations of lonafarnib and passaged 5 times. To initiate passaging, HEp-2 cells (2 × 10^5^) were seeded in a 12-well plate, and both lonafarnib and virus were then added the following day. Lonafarnib was initially added at 500 nM and the virus was added at an MOI of 0.1 per well. Each well was visualized for GFP intensity every 3 days, and 500 µL of the supernatant from the well with GFP was passaged to each well in the next culture plate. The remaining supernatant was stored at –80 °C until further analysis (that is, EC_50_ assay). After 15 days, the supernatant of 5^th^ passage was collected and used to infect freshly seeded cells using the same virus dilution (500 µL) as in all previous passages in the presence of 6 μM of lonafarnib. This procedure was repeated every 3 days until the EC_50_ value observed was greater than 10 fold of the initial EC_50_. The passage culture was set up in triplicate and passaging was performed independently.

### Statistical analysis

The data in the figures represent mean ± SEM. All data were analyzed using GraphPad Prism 8.0 software. Statistical comparison between different groups was performed using corresponding statistical analysis labeled in figure legends combining several experiments. *P*‐values were calculated, and statistical significance was expressed as highly significant with *P < 0.05, **P < 0.01, ***P < 0.001.

### Supplementary information

Supplementary_Materials_RSV fusion_Lonafarnib

PDB Validation Report

## Data Availability

The complete sequence of RSV A2 (GenBank: KT992094), RSV B01 (GenBank: AF013254), RSV ON1 (GenBank: MW582528), and RSV BA9 (GenBank: LC488177) are available on GenBank. The atomic coordinates and structure factor amplitudes of the RSV F protein in complex with lonafarnib have been deposited in the Protein Data Bank with accession codes 8KG5. All materials and reagents generated in this study are available from the corresponding author with a completed Materials Transfer Agreement.
