# Supplementary material for: Farnesyltransferase inhibitor lonafarnib suppresses respiratory syncytial virus infection by blocking conformational change of fusion glycoprotein
Source: Signal Transduct Target Ther. 2024 Jun 10;9:144. doi: 10.1038/s41392-024-01858-5 (PMC11163014; doi:10.1038/s41392-024-01858-5)
Supplement: Supplementary file 1 — Supplementary_Materials_RSV fusion_Lonafarnib [file 41392_2024_1858_MOESM1_ESM.docx]

Supplementary Materials for

Farnesyltransferase inhibitor lonafarnib suppresses respiratory syncytial virus infection by blocking conformational change of fusion glycoprotein

Qi Yang^1, 2, #^, Bao Xue^1, 3,^ ^#^, Fengjiang Liu^1, 2, #^, Yongzhi Lu^1, 4, #^, Jielin Tang^1,2, #^, Mengrong Yan^1, 2^, Qiong Wu^1, 4^, Ruyi Chen^1, 2^, Anqi Zhou^5^, Lijie Liu^1, 2^, Junjun Liu^1, 2^, Changbin Qu^1^, Qingxin Wu^1, 2^, Muqing Fu^6^, Jiayi Zhong^1, 2^, Jianwei Dong^1, 3^, Sijie Chen^5^, Fan Wang^5^, Yuan Zhou^1, 3^, Jie Zheng^7, 8^, Wei Peng^1, 2, *^, Jinsai Shang^1, 4, *^, Xinwen Chen^1, 2, 9, *^

Correspondence to: Wei Peng (peng_wei@gzlab.ac.cn), Jinsai Shang (shang_jinsai@gzlab.ac.cn), Xinwen Chen (chen_xinwen@gzlab.ac.cn)

**This PDF file includes:**

Supplementary figures 1-9

Supplementary table 1

**Supplementary Fig 1. Lonafarnib inhibits RSV-induced syncytium formation. a**, HEp-2 cells were infected with RSV at an MOI of 0.5 for 72 h as photographed. Light micrographs were taken at 10× magnification and syncytia are indicated with black arrows; the boxed regions are enlarged 50× in the right graphs. n = 3 biological replicates. **b**, F proteins of different RSV genotypes were transiently expressed in HEK293T cells. Lonafarnib was diluted to 5 μM and added to the RSV-F expressing cells. The cell-to-cell fusion was observed using microscopy on day 2 after compound addition. n = 3 biological replicates. The results are representatively shown with three random experiments.

**Supplementary Fig 2. Cell viability. a & b**, Cytotoxicity of cyclopamine, jervine, and lonafarnib to HEp-2 cells (**a**), and HBEC (**b**) was measured using CCK8 regent. n = 3 biological replicates. The error bars indicate mean ± SEM. The results are representatively shown with two random experiments.

**Supplementary Fig 3. Experimental chromatogram of DS-Cav1 and D25-Fab. a**, DS-Cav1 was purified using size exclusion chromatogram (SEC) and Coomassie stained SDS-PAGE gel analysis of the purification of DS-Cav1. Lane 1 indicates that DS-Cav1 was purified using affinity chromatography. Lane 2 indicates DS-Cav1 was purified using SEC. **b**, D25-Fab was digested by papain and then purified using SEC. Coomassie stained SDS-PAGE gel analysis of the purification of the digested fragment. Lane 1 indicates the digested fragment was purified using SEC.

**Supplementary Fig 4. Lonafarnib binds the fusion protein of RSV.** SPR sensorgrams DS-Cav1 protein interaction inhibition by a range of lonafarnib concentrations. Global fitting curves are shown as black lines. The vertical dashed lines indicate the transition between the association and dissociation phases. A steady-state affinity approach was used to analyze the data. The results are representatively shown with three random experiments.

**Supplementary Fig 5.** Cryo-EM data processing procedures of lonafarnib bound to the RSV F-D25 Fab complex. **a**, Workflow of the cryo-EM processing steps. **b**, Angular distribution heatmap of all particles used in the final 3D reconstruction. **c**, Fourier shell correlation (FSC) of the final map. **d**, The map is colored according to the local resolution. **e**, 3DFSC histogram of the final map.

**Supplementary Fig 6. HDX-MS probing of lonafarnib binding to the fusion protein of RSV.** Percentage change in HDX uptake (scale at the bottom) is plotted on peptides analyzed in the experiment.

**Supplementary Fig 7. Lonafarnib resistance mutations cluster near a cavity inside pre-fusion F of RSV. a**, Genomic organization of the RSV F signal peptide is colored gray, fragment 2 (F2) is colored blue, peptide 27 (p27) is colored gray, fusion peptide (FP) is colored purple, heptad repeat A (HRA) is colored yellow, heptad repeat B (HRB) is colored goldenrod, fragment 1 (F1) shows amino acid ranging from 137 to 574, the transmembrane domain (TM) is colored pink, and the cytoplasmic tail (CT) is colored cyan. **b**, Structures of an RSV F monomer in the pre-fusion (left) and post-fusion (right) conformation are shown as ribbons. Residues that interact with lonafarnib are shown as red spheres.

**Supplementary Fig 8. *In vivo* efficacy of therapeutic lonafarnib against RSV infection in BALB/c mice. a–f**, BALB/c mice were intranasally inoculated with 1 × 10^6^ PFU RSV A2. At 2 h post infection, the mice were orally administered 17 or 34 mpk lonafarnib for the treatment group (n = 5 mice/group) or vehicle solution only for the control group (n = 5 mice/group) twice daily for 4 days. The mock and vehicle groups were used as negative controls. **a**, Schematic of BALB/c mice infection and treatment. **b**, Mouse body weights were monitored for up to 4 days postinfection. **c**, Mice infected with RSV A2 were killed on day 4  postinfection for the detection of infectious viral titers in the lung tissue using FFA (n = 5). **d–f**, Histological analysis visualizing the virus-induced pathology (**d**) and quantitative scoring of the pathology (**e & f**) in the lung of mice infected with RSV A2 on day 4 postinfection. The analysis was performed as described in **Figs 6d–f**. Each dot represents one mouse at the indicated time point. The data are representative of at least two experiments. The error bars are mean ± SEM. Statistical differences were determined by two-way ANOVA in **b**, **c**, **e** and **f**. **P* < 0.05, ***P* < 0.01; ns, not significant.

**Supplementary Fig 9. Analysis of RSV resistance to lonafarnib.** Wild-type RSV ON1-GFP reporter virus was passaged with increasing concentrations of lonafarnib (from 500 nM to 6000 nM) in HEp-2 cells. EC_50_ of lonafarnib were measured with wild-type virus (RSV ON1-GFP, left) and the 9^th^ passage (RSV ON1-GFP-P9, right) in HEp-2 cells. Data points represent % inhibition by quantifying GFP-positive cells. The error bars indicate mean ± SEM. The results are representatively shown with two random experiments.

Supplementary Table 1. Statistics for data collection, image processing and model building

|  | Lonafarnib bound to RSV F-D25 Fab complex |
| --- | --- |
| **PDB entry** | 8KG5 |
| **EMDB entry** | EMD-37210 |
| **Data collection and processing** | |
| Magnification | 165,000 |
| Voltage (keV) | 300 |
| Electron exposure (e^–^/Å^2^) | 50 |
| Defocus range (μm) | −1.2 to −2.4 |
| Pixel size (Å) | 0.73 |
| Symmetry imposed | C1 |
| Final particle images (no.) | 37,059 |
| Map global resolution (Å) | 3.17 |
| Global resolution FSC threshold | 0.143 |
| Map local resolution range (Å) | 2.8 to ꝏ |
| Local resolution FSC threshold | 0.143 |
| **Refinement** | |
| Map sharpening *B* factor (Å^2^) | −77.6 |
| Model composition | |
| Non-hydrogen atoms | 12,307 |
| Protein residues | 1,577 |
| Ligands | 3 |
| *B* factors (Å^2^) | |
| Protein | 74.38 |
| Ligand | 93.83 |
| R.m.s. deviations | |
| Bond lengths (Å) | 0.008 |
| Bond angles (°) | 1.282 |
| Validation | |
| MolProbity score | 1.94 |
| Clashscore | 11.18 |
| Poor rotamers (%) | 0.49 |
| Ramachandran plot | |
| Outliers | 0.00 |
| Allowed | 5.51 |
| Favored | 94.49 |
